# Image analysis of the intracranial lead bending phenomenon during deep brain stimulation

**DOI:** 10.1371/journal.pone.0237537

**Published:** 2020-08-12

**Authors:** Minsoo Kim, Na Young Jung, Jin Woo Chang

**Affiliations:** 1 Department of Neurosurgery, Samsung Medical Center, Seoul, Korea; 2 Department of Medicine, Graduate School, Yonsei University College of Medicine, Seoul, Korea; 3 Department of Neurosurgery, Ulsan University Hospital, University of Ulsan, College of Medicine, Ulsan, Korea; 4 Department of Neurosurgery, Yonsei University College of Medicine, Seoul, Korea; National Institue on Drug Abuse, UNITED STATES

## Abstract

**Background:**

An accurate and precise surgical procedure is crucial for patient safety and treatment efficacy of deep brain stimulation (DBS).

**Objectives:**

To investigate the characteristics of intracranial lead bending phenomenon after DBS, and to suggest the methods to avoid bending-related complications.

**Methods:**

A retrospective review of brain computed tomography scans after DBS was performed. Using 3-dimensional reconstruction, the maximal distance between the planned trajectory and actual lead location was measured. When the distance exceeded the lead body diameter, the lead was considered bent. The distance between the bending point and planned trajectory, and the relative direction between the bending point and lead securing site were analyzed. Changes over time in the range of lead bending and depth were analyzed when possible.

**Results:**

A total of 190 implanted leads in 102 patients were analyzed; 104 leads (54.7%) were bent. The average deviation of bent leads was 2.3 mm (range, 1.3–7.1 mm). Thirty-five (18.4%) and seven leads (3.7%) had deviations exceeding twice and three times the lead body diameter, respectively. Angles between the deviation point and securing site at the skull ranged from 135–180° in 83 leads (53.2%), 45–135° in 58 (37.2%), and 0–45° in 15 (9.6%). Among 17 leads that were initially bent, 16 had less deviation compared to baseline. The lead depth increased in 35 (92.1%) of 38 leads by 1.2 mm (range, 0.1–4.7 mm).

**Conclusion:**

The extent of lead bending should be considered during the planning and procedural phases of intracranial lead implantation for DBS.

## Introduction

Deep brain stimulation (DBS) is an established treatment modality used for managing various types of drug refractory diseases, including movement disorders and psychiatric disorders [1–6]. Although there is a possible risk of surgical side effects, such as intracerebral hemorrhage, infection, or psychiatric problems, one of the advantages of DBS is the precise preoperative surgical planning and its reproducibility during the procedure. To minimize potential risks, various factors, especially brain structures near the trajectory (e.g., the sulcus/gyrus, vessels, and ventricles) and electricity conductivity, are considered in the planning stage [7–9]. Then, surgeons perform the procedures under the assumption that the procedure is identical to the planned procedure.

**Fig 1:**
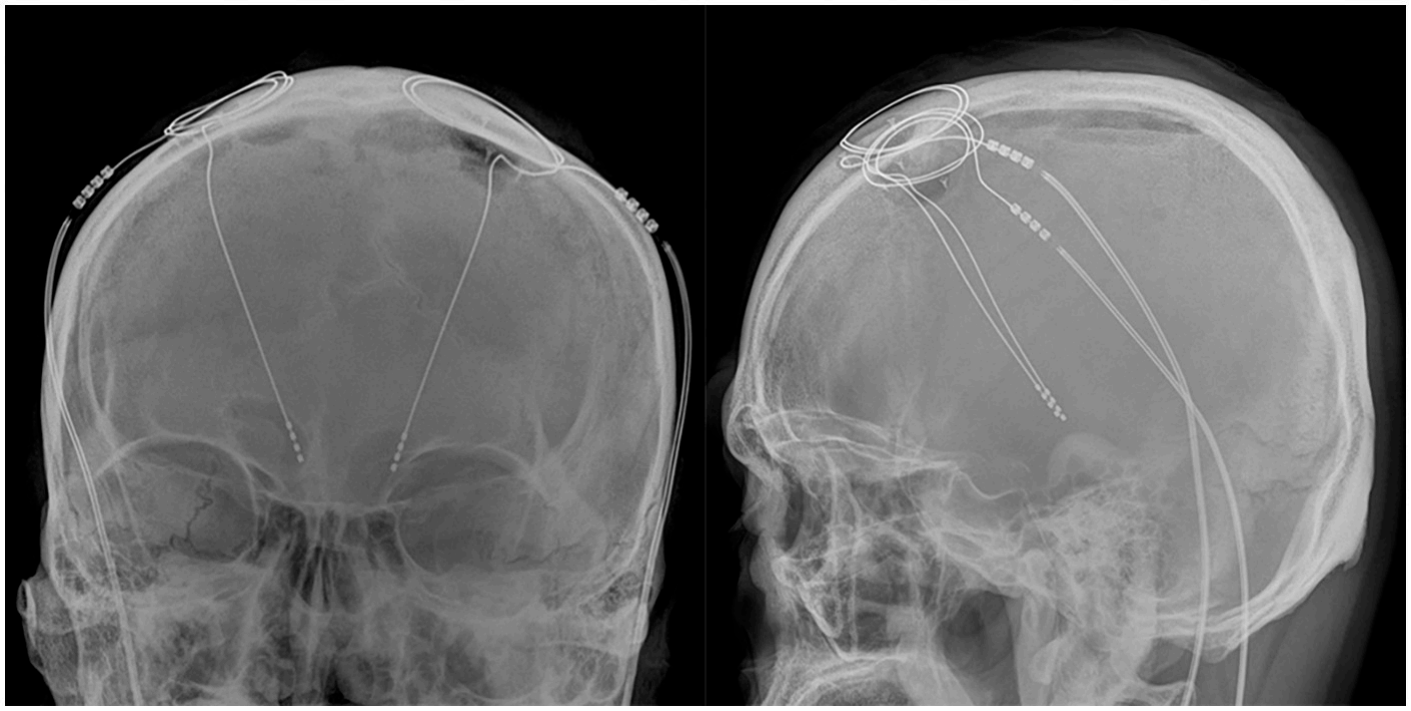
A representative patient’s postoperative intracranial leads on plain radiographs: The leads are obviously bent.

However, even with the best efforts to perform the procedure precisely, deviation of the lead from the planned trajectory [10–12] is often seen, as well as a bent lead (Fig 1). This indicates that the procedure was not identical to the planned procedure, which does not guarantee risk avoidance, as mentioned earlier. We examined whether the intracranial leads actually become bent, and also investigated the characteristics of the lead bending phenomenon and its temporal changes. Moreover, we suggest a possible mechanism of and some methods for avoiding the lead bending phenomenon.

## Materials and methods

### Study design

We retrospectively reviewed postoperative, thin-slice (less than 1.5-mm thickness) brain computed tomography (CT) scans that were taken immediately after the performance of DBS surgery from December 2005 to December 2015 at a single center by a unique surgical team. Patients with inappropriate CT images for analysis due to the motion artifact or thicker image slices; patients who suffered intraoperative complications, such as intracerebral hemorrhages; and patients whose lead trajectory not through the Kocher’s point were excluded. If patients underwent follow-up brain CT after at least 12 months, their follow-up images were also analyzed. This study was approved by the hospital’s institutional review board. Informed consent was waived because of the retrospective nature of the study and the minimal risk to the patients.

### Surgical procedure

Overall, the surgical procedure was performed according to an established standard protocol. Details of the procedure have been described in our previous report [13]. On the day of electrode implantation, each patient was placed in a Leksell G frame (Elekta Instruments, Inc., Stockholm, Sweden), and then a 1.5-Tesla stereotactic magnetic resonance imaging system (Achieva 2.6.3.1, Philips, Best, Netherlands) was used. Surgical targets with a trajectory were planned using the Leksell SurgiPlan software (version 10.1.1, Elekta Instruments, Inc.).

Intracranial lead implantation was performed under local anesthesia. We made a 14-mm diameter burr hole, followed by a small 2–3 mm dural incision to avoid cerebrospinal fluid leakage. Then, the microTargeting Single Insertion Electrode (FHC, Inc., Bowdoin, ME, USA) was inserted through the planned trajectory using the microTargeting Drive System (FHC, Inc.). We inserted a single microelectrode and performed microelectrode recording in all cases. When the permanent location was approved, an intracranial lead (model 3387, Medtronic Inc., Minneapolis, MN, USA) was inserted through the trajectory, and then it was securely fastened with a co-packaged burr-hole lead holding system.

Immediately after electrode implantation, each patient was assessed using a brain CT system (Somatom Sensation 64, Siemens Healthcare GmbH, Erlangen, Germany) to confirm no procedure-related complications and accurate electrode placement according to the institutional protocol for DBS. Then, the implantable pulse generators (Soletra or Activa SC, Medtronic Inc.) were implanted into each patient’s anterior chest wall pocket above the pectoralis major muscle, under general anesthesia.

### Image analysis and processing

**Fig 2:**
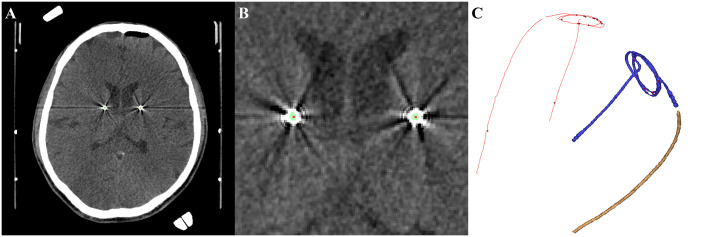
Process of 3-dimensional reconstruction and centerline rendering. A. A postoperative brain computed tomography scan was used in the calculation. B. Green indicates intracranial leads, which were defined as an area of Hounsfield units exceeding 2700, and red indicates the centerline rendered with software logic. C. Reconstructed intracranial lead (right side, volume rendered) and rendered centerline (left side, red).

All images were anonymized after data collection. The images were then analyzed using commercially available software, Mimics Medical (version 17.0, Materialise NV, Leuven, Belgium). As the range of Hounsfield units was more than 2700, the images were 3-dimensionally (3-D) reconstructed. The exact center of the selected 3-D lead structure was rendered using software logic. The image processing is shown in Fig 2.

### Bending phenomenon and its characteristics

**Fig 3:**
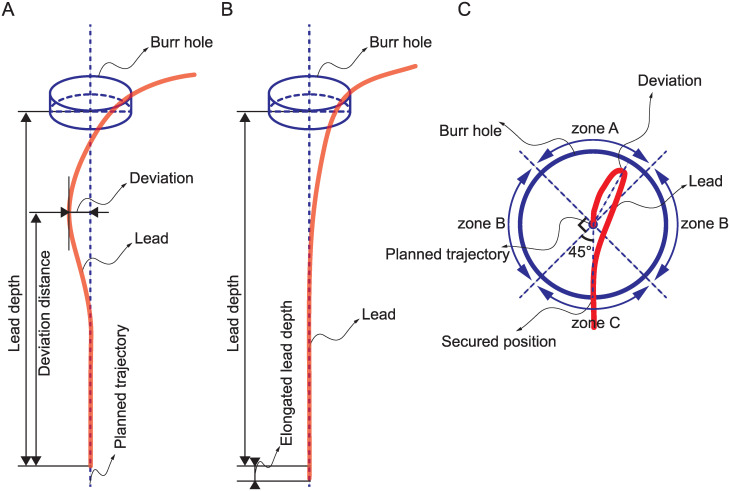
Definition and description of the variables. A. Concept of lead bending immediately after surgery. B. The bent lead was gradually straightened, and the lead depth increased as the lead was locked at the burr hole. C. Drawing of the direction of bending perpendicular to the planned trajectory (center of the circle). The deviated area (zones A, B, and C) were determined when the lead locking position was regarded as 0°.

The distance between the lead tip and skull inner surface was defined as the lead depth. Since the bending phenomenon seems to occur at a distal portion from the tip, we assumed that the proximal part (about 30 mm from the tip) was straight, *i*.*e*., identical to the planned trajectory. Then, an imaginary line extending that straight line was rendered. The distance between the lead and imaginary line, *i*.*e*., deviation, was evaluated using the centerline, as aforementioned. When the deviation was greater than the diameter of the lead, we assumed that the bending phenomenon occurred (Fig 3A). The distance from the lead tip and deviation was defined as the deviation distance. When deviation was not observed, the lead was considered straight (Fig 3B).

### Direction of bending

To determine the direction of deviation, the angle between two vectors, as well as the bending and lead secured positions at the burr hole, were measured in the perpendicular plane to the planned trajectory (Fig 3C). The angle was categorized as zones A (135–180°), B (45–135°), or C (0–45°). Zone A refers to the opposite direction of the lead locking position when we set the planned trajectory at center; zone B means both sides; and zone C is the same direction as the lead locking position.

### Changes in lead bending over time

In cases with follow-up brain CT scans over a 12-month period, the same aforementioned measurement protocol was used. The number of patients with bending, as well as changes in deviation and depth of the electrode, were measured.

### Statistical analysis

The deviation, deviation distance, and direction of bending were expressed using descriptive statistics such as a percentage, mean, and range. Differences between groups were analyzed using the Kruskal-Wallis test and paired t-test. *P*-values <0.05 were considered statistically significant. All data analyses were conducted using SPSS software (version 23, IBM Corp., Armonk, NY, USA).

## Results

### Patient demographics

A total of 102 two patients (56 men and 46 women) with 190 electrodes were analyzed in this study. The number of patients who underwent bilateral and unilateral implantation were 88 and 14, respectively. The mean age of patients was 54 years (range, 14–77 years). The reasons for DBS surgery were as follows: 56 patients had Parkinson disease, 20 had dystonia, 17 had essential tremor, and nine had other reasons. Patients with CT images that are inappropriate for analysis (n = 26), postoperative hemorrhagic complication (n = 1), and lead insertion through trajectory other than the Kocher’s point (n = 1) were excluded. Twenty-one patients with 38 electrodes were followed up through brain CT after at least 12 months postoperatively. All implanted electrodes were model 3387, which had a body diameter of 1.27 mm.

### Changes in lead bending

**Fig 4:**
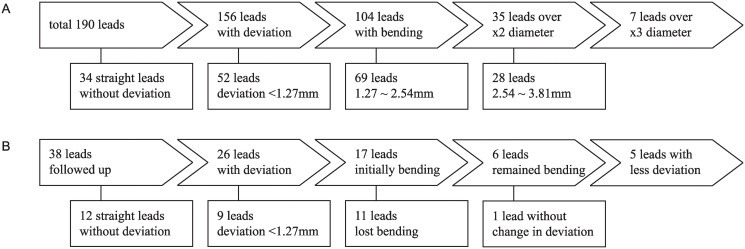
Flow chart of intracranial leads. A. Deviation of intracranial leads immediately after surgery. B. Temporal changes.

Among 190 leads, 156 showed signs of deviation (Fig 4A), and 104 (54.7%) were determined to be bent with a deviation over the lead diameter of 1.27 mm. The other 34 leads (17.9%) were initially considered straight, without signs of deviation. The average deviation of the bent leads was 2.3 mm (range, 1.3–7.1 mm; standard deviation, 0.9). Thirty-five leads (18.4%) had a deviation more than twice the electrode diameter (2.5 mm), and seven (3.7%) had a deviation more than triple the electrode diameter (3.8 mm).

### Direction of bending

Eighty-three (53.2%) of 156 deviated leads were categorized as zone A (more than 135°). Fifty-eight (37.2%) and 15 (9.6%) leads were categorized as zone B (45–135°) and zone C (0–45°), respectively. There were no differences in deviation among zones A, B, and C (*p* = .358, Kruskal-Wallis test).

### Changes on follow-up brain CT scans

Twenty-one patients with 38 leads had a follow-up brain CT scan after at least a 12-month postoperative period (Fig 4B). Seventeen leads were determined to be bent on initial brain CT scan, six of which remained bent on follow-up CT. Among the initially bent 17 leads, 16 had less deviation, and 11 were no longer bent. The depth of the leads, *i*.*e*., the length of the electrode, was elongated in 35 leads (92.1%) by an average of 1.2 mm (range, 0.1–4.7 mm; standard deviation, 1.0) and shortened in two leads (5.3%) by 1.4 mm and 0.7 mm; no change in depth was observed in only one lead (2.6%). Eight leads had a depth of elongation of more than 1.5 mm. Elongation of more than 3 mm, *i*.*e*., the length of the electrode and spacing, was prominent in two leads (2.6%). The average change in the electrode depth among all of the leads was 1.0 mm (range, -1.4–4.7 mm).

In 12 cases that did not initially have a deviation, the change in lead depth was not different than that of leads with deviation (mean, 1.2 mm; range, -0.7–4.7 mm; *p* = .642). All data of this study are available in S1 File.

## Discussion

**Fig 5:**
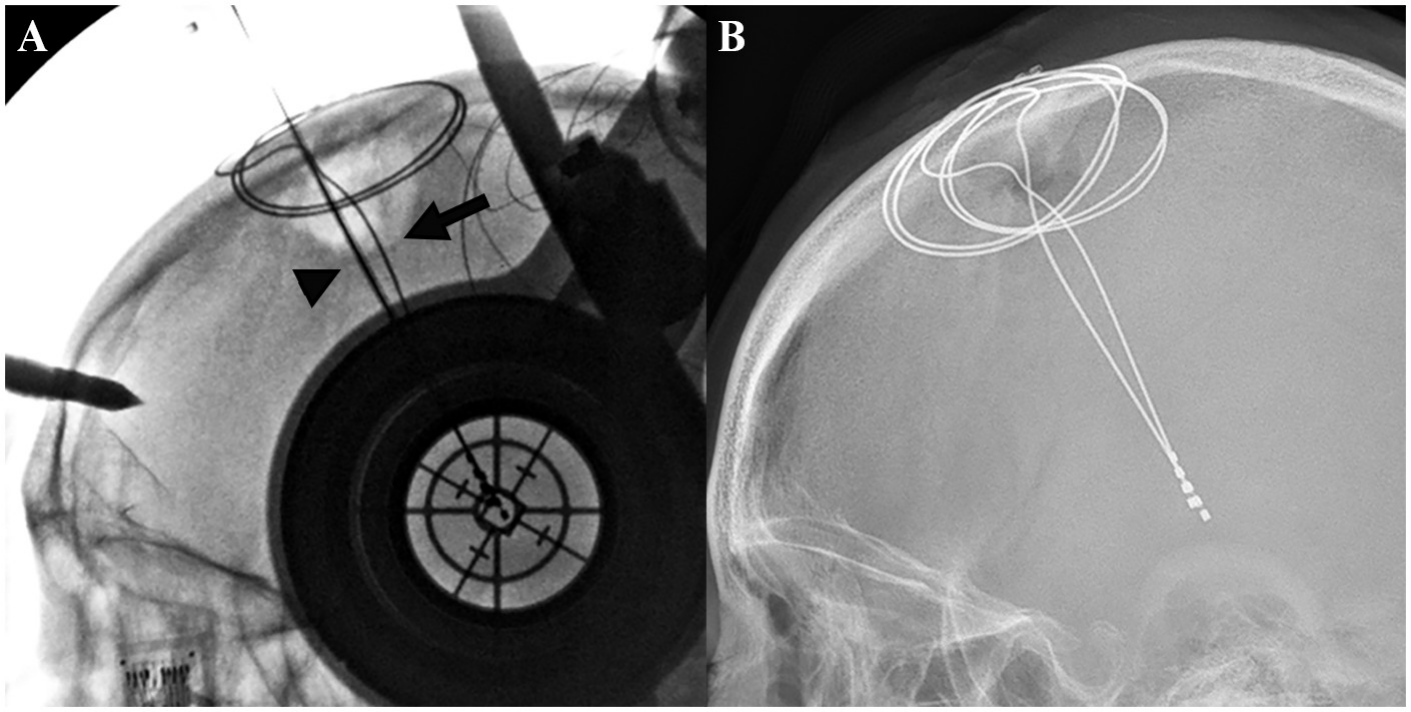
Timing of bending occurrence. A. When the implanted lead is still secured in the guide tube, the lead is straight (arrowhead). In contrast, another lead which is permanently implanted and secured by the burr hole cover is already bent (arrow). B. Immediate postoperative lateral skull X-ray of the identical patient shows that both leads are bent.

**Fig 6:**
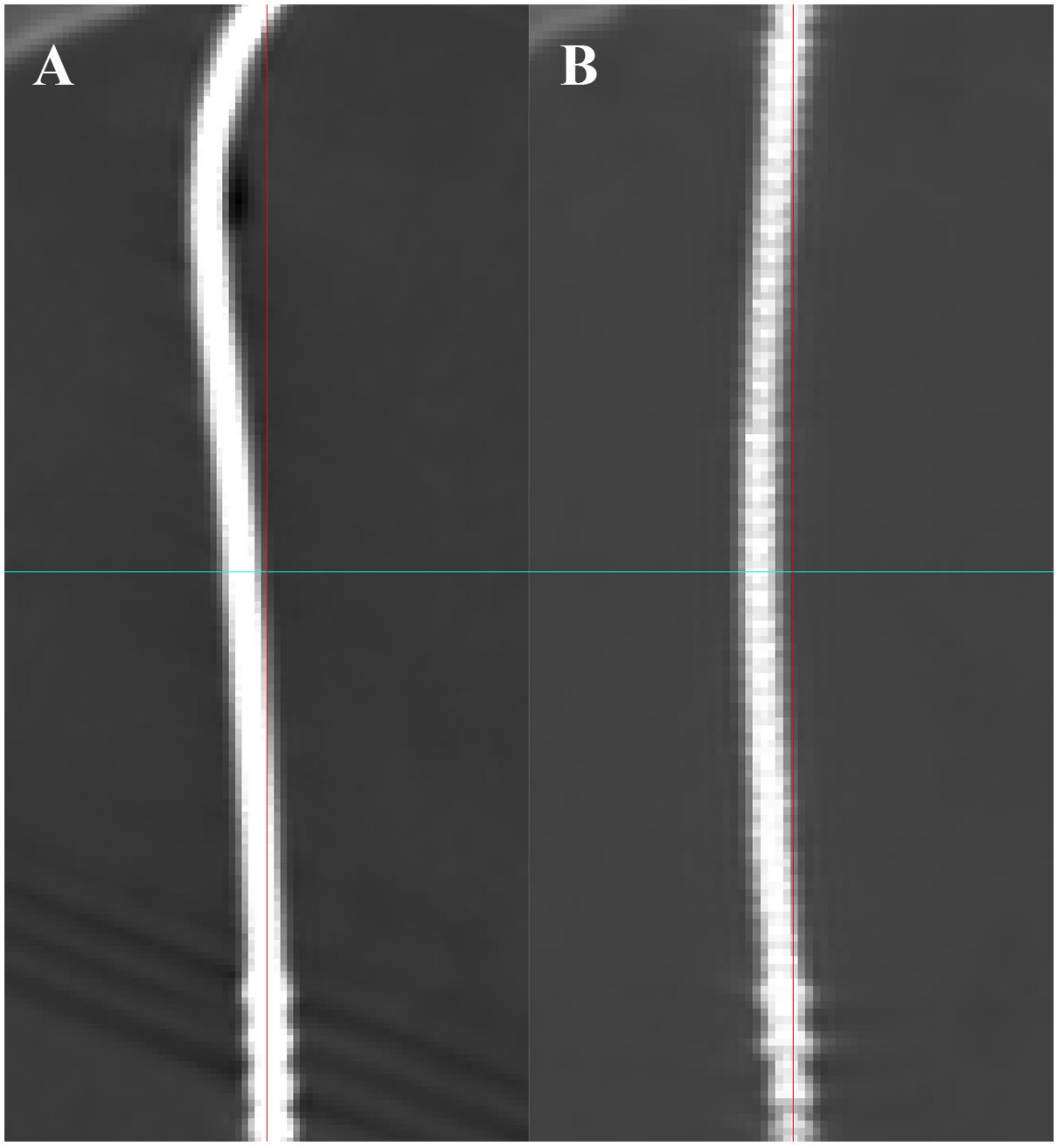
Change of lead shapes over time. These figures demonstrate how the lead shapes change over time. A lead that was bent on the day of operation (A) became straightened after 24 months (B).

The results of this study showed that DBS leads become bent during the surgery (Fig 5). More than 50% of implanted leads had deviation that was more than the lead body diameter (1.73 mm). Some of the leads (18.4%) even had deviation more than twice the lead body diameter. Operators do not think these ranges of deviation occurred during the procedure. Moreover, the depth of the leads deepened as the bending phenomenon was relieved over time. As the lead length deepens, the electrode contacts also migrate along the trajectory (Fig 6). According to the manufacturer’s specification, the lead model 3387 has an electrode length of 1.5 mm and spacing of 1.5 mm between electrodes. Other commercially available devices, such as model 3389 (Medtronic Inc.) and some within the model 6100 series (Saint Jude Medical, Little Canada, MN, USA) or Vercise Cartesia series (Boston Scientific, Marlborough, MN, USA), have shorter electrode spacing than the one we used, and these devices can induce a very significant clinical response in patients. The results of our study showed that electrode migration consequently stimulates areas that are not intended to be stimulated. A migration of 3 mm means that the next electrode is currently contacting the initially stimulated brain area. These findings indicate that DBS leads are not implanted in the same place as the one initially planned by surgeons.

Although the data is not currently available, the authors suggest that the lead bending phenomenon can be a source of the patient’s changes in motoric symptoms, neuropsychiatric condition, or even complications that need surgical intervention. As is well-known, electrical stimulation in deeper areas may induce unwanted responses, such as the following: A. phosphenes, a visual symptom due to optic tract irritation in patients with globus pallidus pars interna stimulation; B. mood changes, akinesias, or muscle contractions from the stimulation of the substantia nigra or the internal capsule in subthalamic nucleus stimulation; and C. ataxia, paresthesia, dysarthria, or muscle contractions from unexpected irritation of cerebellar fibers, medial lemniscal pathway, or internal capsule in patients whose leads are implanted in ventral intermediate nucleus. Therefore, clinicians should consider the chance of lead deepening in patients whose response to stimulation does not correlate with the findings at the timing of surgery.

The lead bending phenomenon may be harmful for patients. The advantages of stereotactic procedures, such as DBS, depth electrode insertion, and stereotactic biopsy, are their minimal invasiveness and maximal safety. When planning the surgical trajectory, we pay careful attention to avoid risky structures, such as the vessel and sulcus. However, we often inevitably place these critical structures away from the planned trajectory by no more than a few millimeters during planning. Bending of the lead toward those risky structures seems likely to induce tension on the vessels or brain tissues, which may consequently result in critical procedure-related complications, such as a hemorrhage. Moreover, air trapping near an electrode on a brain CT scan was frequently visible near the location of deviation. However, we did not analyze the air-trapping phenomenon.

We assume that the bending phenomenon happens during lead locking at the burr hole; other studies have reported a similar assumption [14–16]. Conventionally, the lead is locked with a plastic-like or rubber-like material that caps the burr hole; this kinks the lead at a corner, and thus this may somewhat elongate the deepening during the lead securing procedure. Repetitive pushing and pulling to locate the lead at a precise depth can cause lead bending. The angle between the deviation and locking may support this hypothesis, as the lead’s resistance to push and pull gives vector to bending instead of deepening.

We suggest some possible solutions to minimize the occurrence of the bending phenomenon that may help prevent procedure-related complications. First, when surgeons lock the lead at the skull, it seems better to avoid the opposite direction of where critical structures, which may be injured by a bent lead, are lying. Securing the leads in the direction of a risky structure is beneficial. Second, it may be helpful to use commercially available devices that fix the electrode at the center of the burr hole to prevent the electrode from deepening. For example, the manufacturer’s supply devices such as the Stimloc (Medtronic Inc.), Guardian burr hole cover (Saint Jude Medical), and SureTek burr hole cover (Boston Scientific). Based on this perspective, adjusting the center of the burr hole to make it identical to the planned trajectory can also be a possible solution.

This study had several limitations. First, the present study was a retrospective review of patients’ imaging studies; therefore, it was limited in term of sample availability and the type of information collected. Second, although all of the postoperative CT scans were obtained within a few hours after intracranial lead implantation, follow-up imaging studies were not obtained during a specific timespan. Third, only some of the patients underwent follow-up imaging studies, and there were varying levels of clinical significance; therefore, changes in the bending phenomenon over time seem to not have been evaluated properly. Lastly, only a few CT scans showed massive air trapping, and complications were excluded from this study; therefore, a possible relationship between lead bending and complications could have been overlooked.

The lead bending phenomenon can be a minor and meaningless issue during the entire surgical procedure. Since the brain is somewhat movable and resistant to shifting, a small amount of displacement by a thin intracranial DBS lead may have minimal effect. However, we think that since the lead is thin, the brain is more easily injured by shearing force. Since we have not yet performed a precisely modeled test, a further study is required.

Intracranial leads used in DBS may become bent during implantation. Also, the range of bending can somewhat exceed the operator’s expectation. As this can be a source of treatment-related complications, the operator should consider the possibility of lead bending and use avoidance strategies.

## Supporting information

S1 File (XLSX)
